# MANAGEMENT OF ENDOCRINE DISEASE: Imaging for the diagnosis of malignancy in incidentally discovered adrenal masses: a systematic review and meta-analysis

**DOI:** 10.1530/EJE-16-0461

**Published:** 2016-08-01

**Authors:** Jacqueline Dinnes, Irina Bancos, Lavinia Ferrante di Ruffano, Vasileios Chortis, Clare Davenport, Susan Bayliss, Anju Sahdev, Peter Guest, Martin Fassnacht, Jonathan J Deeks, Wiebke Arlt

**Affiliations:** 1 Institute of Applied Health Research; 2 Institute of Metabolism and Systems ResearchUniversity of Birmingham, Birmingham, UK; 3 Division of EndocrinologyMetabolism, Nutrition and Diabetes, Mayo Clinic, Rochester, Minnesota, USA; 4 Department of ImagingSt Bartholomew’s Hospital, Barts Health, London, UK; 5 Department of RadiologyQueen Elizabeth Hospital, University Hospital Birmingham NHS Foundation Trust, Birmingham, UK; 6 Department of Internal Medicine IDivision of Endocrinology and Diabetes, University Hospital Würzburg, University of Würzburg, Würzburg, Germany; 7 Comprehensive Cancer Center MainfrankenUniversity of Würzburg, Würzburg, Germany; 8 Centre for EndocrinologyDiabetes and Metabolism, Birmingham Health Partners, Birmingham, UK

## Abstract

**Objective:**

Adrenal masses are incidentally discovered in 5% of CT scans. In 2013/2014, 81 million CT examinations were undertaken in the USA and 5 million in the UK. However, uncertainty remains around the optimal imaging approach for diagnosing malignancy. We aimed to review the evidence on the accuracy of imaging tests for differentiating malignant from benign adrenal masses.

**Design:**

A systematic review and meta-analysis was conducted.

**Methods:**

We searched MEDLINE, EMBASE, Cochrane CENTRAL Register of Controlled Trials, Science Citation Index, Conference Proceedings Citation Index, and ZETOC (January 1990 to August 2015). We included studies evaluating the accuracy of CT, MRI, or ^18^F-fluoro-deoxyglucose (FDG)-PET compared with an adequate histological or imaging-based follow-up reference standard.

**Results:**

We identified 37 studies suitable for inclusion, after screening 5469 references and 525 full-text articles. Studies evaluated the accuracy of CT (*n*=16), MRI (*n*=15), and FDG-PET (*n*=9) and were generally small and at high or unclear risk of bias. Only 19 studies were eligible for meta-analysis. Limited data suggest that CT density >10HU has high sensitivity for detection of adrenal malignancy in participants with no prior indication for adrenal imaging, that is, masses with ≤10HU are unlikely to be malignant. All other estimates of test performance are based on too small numbers.

**Conclusions:**

Despite their widespread use in routine assessment, there is insufficient evidence for the diagnostic value of individual imaging tests in distinguishing benign from malignant adrenal masses. Future research is urgently needed and should include prospective test validation studies for imaging and novel diagnostic approaches alongside detailed health economics analysis.

## Introduction

An incidentally discovered adrenal mass is a frequent occurrence, serendipitously discovered in around 5% of cross-sectional abdominal imaging carried out for purposes other than a suspected adrenal problem (1, 2, 3). Due to the increasingly widespread use of cross-sectional imaging, adrenal incidentalomas represent a significant challenge to health care budgets. The rates of computed tomography (CT) scans carried out in the USA soared from 3 million per annum in 1980 to 81.2 million in 2014 (4). Concurrently, in the UK, 5 million CT scans were undertaken in 2012/2013, increasing from 1 million in 1996/1997 (www.england.nhs.uk/statistics/statistical-work-areas/diagnostics-waiting-times-and-activity/imaging-and-radiodiagnostics-annual-data/). The use of repeated and multiple modality imaging in adrenal incidentalomas represents a major challenge to health care budgets and a burden to patients affected. Therefore, evidence-based guidance on the use of imaging in adrenal incidentalomas is urgently needed.

Prevalence of adrenal incidentalomas increases with age (3% at 40 years, 10% at 70 years) (5), and is very low in children (<0.5%) (6). A key consideration for the ­diagnostic workup of adrenal incidentalomas is whether the adrenal mass is hormone-producing, requiring exclusion of pheochromocytoma, Cushing syndrome, and, in hypertensive patients, primary aldosteronism. Second, and usually perceived as most important by the affected patient, the possibility of malignancy has to be considered.

In patients with a history of extra-adrenal malignancy, the detection of a new adrenal mass raises suspicion of metastasis, but also requires careful exclusion of other causes. In cancer patients, the likelihood of an adrenal nodule being malignant is approximately 20%; eventually, only 70% of adrenal lesions surgically removed on the basis of imaging results are confirmed as metastasis by histology (7, 8, 9).

While the detection of adrenal metastasis is a rarity in adrenal incidentaloma patients who do not have a history of extra-adrenal malignancy, the discovery of an adrenocortical carcinoma (ACC) is not uncommon. Larger clinical and surgical adrenal incidentaloma series report an ACC prevalence of 1.4–12% (2, 10, 11, 12), with variability mostly driven by referral bias. Radiological studies describe lower rates of malignant and functionally active adrenal tumors, but usually lack uniform endocrine evaluation and an optimal reference standard such that malignant lesions could be missed (3).

An adrenal incidentaloma is most frequently noted on CT or MRI scans carried out for other purposes. Both ­imaging modalities can assess the lipid content in the adrenal mass, which serves as the basis for differentiating between a benign (high lipid content) and a potentially malignant (low lipid content) adrenal mass. However, at least a third of benign adrenal adenomas have been shown to be lipid-poor (13, 14). This lack of specificity causes many patients to undergo multiple scans and imaging modalities, often followed by surgery, with histology ultimately revealing a benign mass that would not have required surgery in 30–55% of patients (2, 15).

In addition to the general radiological criteria of size of the mass and its appearance (heterogeneity, ­borders, ­invasion) (13, 16), multiple imaging parameters are employed for the differential diagnosis of adrenal incidentaloma. These include unenhanced CT with assessment of tumor density, contrast-enhanced timed washout CT studies, MRI chemical shift analysis, and, more recently, ^18^F-fluoro-deoxyglucose (FDG)-PET (FDG-PET) in combination with CT (PET-CT).

However, despite their widespread use in the workup of adrenal incidentalomas, the optimal choice, sequence and performance of imaging tests to distinguish benign from malignant adrenal masses is unclear (17), and clinical practice remains more expert-based than ­evidence-based. Individually, published reports are often unconvincing due to small sample sizes, heterogeneity of included populations and different imaging techniques or cut-offs as well as poor reference standards. Due to this, many patients with adrenal tumors undergo multiple scans, annual follow-up imaging and even unnecessary surgery (2), with previous guidelines and reviews requesting annual follow-up imaging for up to 2years in most adrenal incidentaloma patients not undergoing surgery (16, 17, 18).

We have carried out a systematic review and meta-analysis of the diagnostic performance of imaging tests in incidentally discovered adrenal masses, with the aim of facilitating evidence-based recommendations on the effective use of imaging in adrenal incidentalomas. With advances in the evaluation of diagnostic test accuracy increasing the awareness of potential sources of bias (19, 20, 21, 22), as well as summarizing study findings, we provide insights into the validity and applicability of the available evidence base and identify current limitations.

## Methods

This review follows methods as set out in the Cochrane Handbook for Systematic Reviews of Diagnostic Test Accuracy (23) and reporting standards set in the Preferred Reporting Items for Systematic Reviews and Meta-analysis (PRISMA) statement (24). This paper reports on the accuracy of CT, MRI, and FDG-PET or PET-CT at commonly used thresholds for the diagnosis of malignant adrenal masses in individuals with incidentally identified lesions, including those identified in individuals with known malignancy.

### Data sources and searches

MEDLINE (Ovid), MEDLINE In Process (Ovid), EMBASE (Ovid), Cochrane CENTRAL Register of Controlled Trials and Cochrane Database of Systematic Reviews, Science Citation Index, and Conference Proceedings Citation Index (Web of Science) and ZETOC (British Library) databases were searched by an Information Specialist (SB) for titles published between 1990 and 13 August 2015. Studies published before 1990 were not considered to be representative of current imaging technologies. The full search strategy as designed for MEDLINE is available in Supplementary Table 1, see section on supplementary data given at the end of this article. The reference lists of included studies and relevant systematic reviews were reviewed for additional eligible studies.

### Study selection

We considered all studies of CT, MRI, or FDG-PET in adult participants with incidentally identified adrenal masses for inclusion. These included both patients in whom imaging for any indication other than an adrenal mass led to the detection of an adrenal mass (true adrenal incidentalomas) and patients with an adrenal mass detected by imaging carried out for staging or follow-up of extra-adrenal malignancy. Studies that did not report the original indication for imaging are reported, but were not included in the meta-analyses.

The target condition of interest was the detection of adrenocortical carcinoma (ACC) or adrenal metastases from an extra-adrenal primary malignancy. We included all studies with reference standards where i) at least 50% of participants with ACC or a malignant adrenal mass had a histologically proven reference standard diagnosis (obtained either through adrenalectomy or adrenal biopsy) and ii) at least 50% of those with a benign adrenal mass had their final diagnosis reached by either histology or imaging-based follow-up of any duration.

In collaboration with clinical and radiological experts from the European Society of Endocrinology (ESE) and European Network for the Study of Adrenal Tumors (ENSAT) Clinical Practice Guideline Committee for the management of adrenal incidentalomas, we selected five commonly used diagnostic imaging thresholds for inclusion: (i) non-contrast CT: tumor density measured in Hounsfield units (HU) >10; (ii) contrast-enhanced CT washout studies: absolute percentage washout (APW) and/or relative percentage washout (RPW) at any washout percentage or delay time on enhanced CT; (iii) MRI chemical shift analysis: loss of signal intensity between in and out of phase images (including both qualitative and quantitative estimates of signal loss); and, for FDG-PET or PET-CT, (iv) the maximum standardized uptake value (SUV_max_); and (v) the ratio of SUV_max_ in the adrenal gland compared with the liver (adrenal liver ratio (ALR)).

We excluded studies where more than half of participants presented with endocrine symptoms, or were otherwise suspected of hormone excess, and those concerned with the diagnosis of adrenomedullary tumors; pheochromocytomas can usually be detected by measuring plasma or urinary metanephrines and their imaging characteristics overlap with those observed in adrenocortical malignancy and adrenal metastases. Therefore, studies with more than 30% pheochromocytomas in the disease-positive group were excluded, unless data could be disaggregated to allow their exclusion from the analysis. We also excluded studies in pediatric populations, sample size <10, data collection before 1990, and with insufficient data presented to allow the construction of a 2×2 diagnostic contingency table. Non-English language studies and studies only reported in conference abstracts were excluded.

Title and abstract screening and full-text inclusion was carried out independently by two reviewers (I B, J Di,). Any disagreements were resolved through discussion or referral to a third reviewer (C D, V C, L F R).

### Data extraction and quality assessment

Data extraction was carried out independently by at least two authors (I B, J Di, L F R, V C, C D) using a standardized and piloted data extraction form. Details of the study design, participants, lesion characteristics, index test(s) or test combinations and index test positivity thresholds, reference standards, and 2×2 diagnostic contingency table data were extracted. Any malignant masses detected in addition to ACC or adrenal metastases (malignant pheochromocytomas, other malignant medullary tumors or other malignancy) were considered disease positive, as their clinical management is sufficiently similar. If study data could not be fully disaggregated, the malignant group could include up to 10% benign masses and up to 10% of the benign group could include medullary tumors (pheochromocytomas, neuroblastoma, ganglioneuroma, or schwannoma). Discrepancies in data extraction were resolved by consensus or by a third reviewer.

We considered the risk of bias and concerns about the applicability of findings related to the patients, tests, reference standard, and execution of each study, using the QUADAS-2 checklist (19), tailored to the review topic. Three authors (I B and V C plus J Di or L F R) independently rated each study with disagreement resolved by consensus.

Patient selection was regarded at risk of bias if consecutive or random selection was not used, patients were selected according to presence of adrenalectomy data, or patients were inappropriately excluded based on previous lesion assessments. Test and reference standard implementation were considered at risk of bias when each was undertaken with knowledge of the other, or when test thresholds were not prespecified, and when final diagnoses of malignancy were not all based on histology or tumor sampling was inadequate, or benignity was assumed without histology or <12 months imaging follow-up. Non-blinded interpretation of other imaging tests added to bias in test interpretation. Risk of bias in the execution of the study was considered when reference standards were not undertaken in all patients, when participants were excluded from analyses, when the reference standards used in malignant or benign cases varied, or when there was no follow-up of suspected benign cases within 6 months.

Concerns about applicability were noted for participants when <90% were recruited with incidentally discovered adrenal tumors or having known or prior malignancy; for tests, when inadequate detail of the test measure was given to allow replication or standard thresholds were not used; and when the reference standard did not allow full disaggregation of the tumor types into malignant and benign.

### Data synthesis and analysis

Data synthesis focused on estimating the accuracy of each test for diagnosis of malignancy for separate clinical pathways for (i) adrenal incidentaloma, that is, investigation of an adrenal tumor detected by imaging carried out for an indication other than suspected adrenal disease and for (ii) history of extra-adrenal malignancy, that is, imaging evaluation or staging in patients with known or prior non-adrenal malignancy. It was considered possible that the accuracy of each test may differ between these clinical pathways. Each study was characterized according to whether the majority (>50%) or nearly all (>90%) individuals were recruited in each pathway, and separate analyses were undertaken for each group. Studies that did not meet these criteria or where the reasons for imaging could not be ascertained were excluded from the analysis. For analysis of MRI chemical shift we restricted inclusion to studies using 1.5 Tesla machines, which were the majority.

Estimates of sensitivity and specificity and 95% CIs for the detection of malignancy were calculated using the binomial exact method when there was only one study, or when there were no false negatives or false positives. Otherwise, the bivariate hierarchical model was used to obtain meta-analytical estimates of average sensitivity and specificity (25). Where possible, the model included terms for random effects for sensitivity and specificity and their correlation, but was simplified when inadequate numbers of studies were available (26).

## Results

### Characteristics of included studies

**Figure 1:**
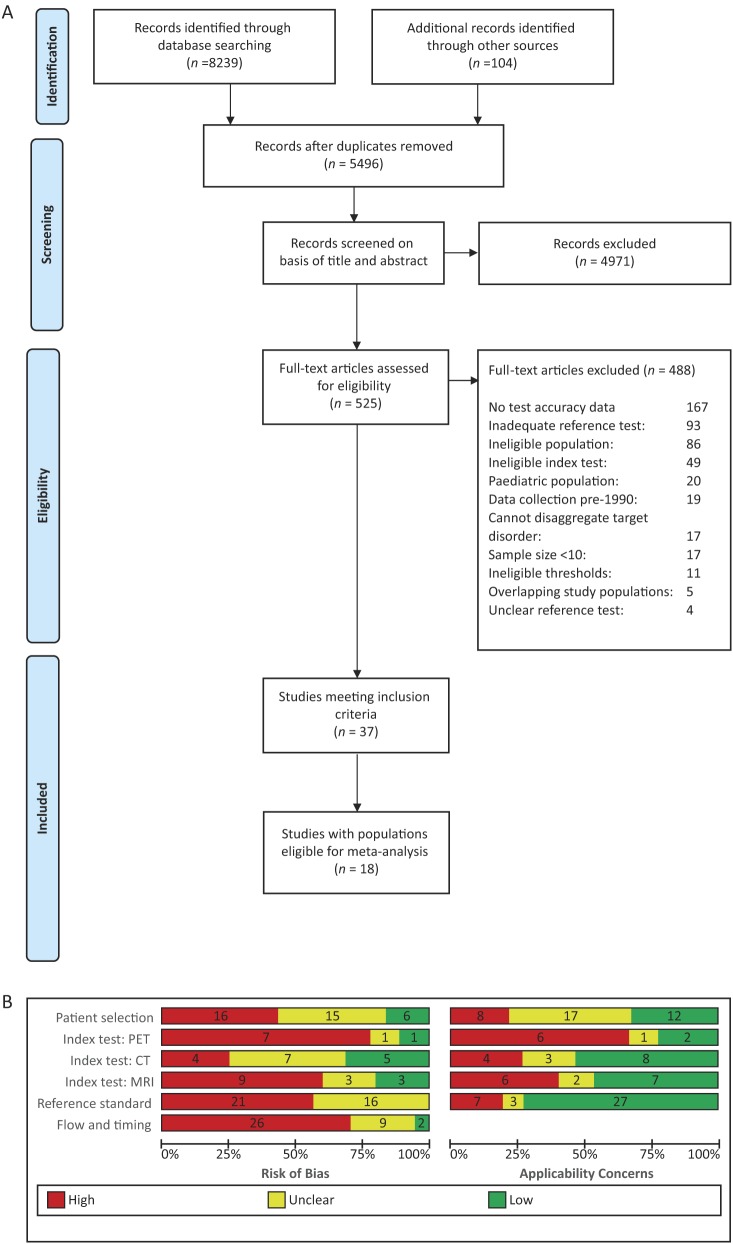
(A) PRISMA flow diagram (adapted from Moher 2009 (24)). (B) Summary risk of bias and concerns about applicability (based on adapted QUADAS-2 (19)).

A total of 5496 unique references were identified and screened for inclusion. Of these, 525 full-text papers were reviewed and 37 studies were included (Fig. 1A) (7, 27, 28, 29, 30, 31, 32, 33, 34, 35, 36, 37, 38, 39, 40, 41, 42, 43, 44, 45, 46, 47, 48, 49, 50, 51, 52, 53, 54, 55, 56, 57, 58, 59, 60, 61, 62). Studies were primarily excluded due to lack of test accuracy data (167 studies), inadequate reference standards (93 studies), and ineligible populations (86 studies). A further 17 studies did not present their data in accordance with our review question and it was not possible to disaggregate their results to allow their inclusion (i.e. >30% pheochromoytomas in the malignant group (*n*=8), >10% medullary tumors (*n*=6) or any malignant mass (*n*=2) in the benign group, and >10% benign masses in the malignant group (*n*=1)) and 11 studies were excluded as they did not use any of our preselected diagnostic thresholds (Supplementary Table 2).

Summary study characteristics are presented in Table 1. CT was evaluated in 16 studies (non-contrast CT was evaluated in 13 studies, contrast-enhanced CT washout studies in 6 studies), MRI in 15 studies, and PET in 9 studies. Studies were generally small with a median sample size of 45 (range 12–181) and less than a third were prospective in design (*n*=10, 27%). Mean prevalence of malignancy was 38% (range 13–74%). Most were conducted in Europe (*n*=15, 41%) and North America (*n*=12, 32%). Datasets for a single imaging test were included from most studies (*n*=34, 92%) compared with a reference standard of histology alone (*n*=14, 38%; excision or biopsy sample) or a mixed reference standard of histology or imaging follow-up (*n*=14, 38%). Reported follow-up periods ranged from 6 to 24 months. Of the papers ­reporting participant recruitment dates (*n*=27, 76%), most were conducted between 2000–2005 (*n*=12, 32%) and 2005–2009 (*n*=9, 24%).

**Table 1:** Summary of the characteristics of the 37 studies fulfilling the inclusion criteria.

**Characteristic**	**Studies (*n*)**	**(%)**
Study design		
Prospective case series	9	(24)
Retrospective case series	19	(51)
Diagnostic case–control (two-gate series)	4	(11)
Design unclear	5	(14)
Population characteristics		
Sample size (participants)	*50.4	^†^(12–181)
Sample size (lesions)	*52.3	^†^(14–146)
Prevalence of malignancy (%)	*38.1	^†^(13–74)
Mean age (years; 29 studies)	^‡^*55.8	^†^(44.1–66.7)
Female participants (%; 31 studies)	*49.4	^†^(6–87)
Mean tumor size (mm; 24 studies)	^‡^*41.9	^†^(22–68.1)
Mean % symptomatic participants (5 studies)	*36	^†^(26–47)
Confirmed hormone excess (%; 11 studies)	*36.3	^†^(0.02–88)
Index tests and thresholds		
CT	16	(43)
Non-enhanced tumor density	*13*	*(*35*)*
Contrast-enhanced washout studies	*6*	*(*16*)*
MRI	15	(41)
Chemical shift loss of signal intensity	*8*	*(*22*)*
Adrenal to liver ratio signal intensity	*8*	*(*22*)*
Adrenal to spleen ratio signal intensity	*5*	*(*14*)*
Adrenal to muscle ratio signal intensity	*2*	*(*5*)*
PET	4	(11)
SUV_max_	*3*	*(*8*)*
SUV_max_ adrenal to liver ratio	*5*	*(*14*)*
PET-CT	5	(14)
SUV_max_	*3*	*(*8*)*
ALR SUV_max_	*4*	*(*11*)*
Population grouping for analysis		
Initial finding incidental in ≥90% included participants	3	(8)
Initial finding incidental in 50–90% included participants	4	(11)
Initial indication for imaging due to known cancer in ≥90% included participants	9	(24)
Initial indication for imaging due to known cancer in 50–90% included participants	2	(5)
Initial finding incidental in <50% OR <50% imaging indication known cancer	2	(5)
Population composition not reported	17	(46)
Reference standard		
Histology alone	16	(43)
Histology and imaging follow-up	15	(41)
Histology and imaging follow-up, plus other reference	5	(14)
Histology plus other reference	1	(3)

SUV_max_, maximum standardized uptake value; ALR SUV_max_, ratio of SUV_max_ in the adrenal gland compared with the liver.

* Mean;

† Range;

‡ Mean of reported means.

Where reported, study populations were highly varied, with only 7 studies (19%) including a majority of participants with purely incidental findings and 11 (29%) focusing primarily on participants with known extra-adrenal malignancy (>50% of population) (Table 1). Studies variously excluded masses with particular imaging characteristics including CT HU<10 (*n*=3), size <10mm (*n*=11), pheochromocytomas (*n*=15), or functioning masses (*n*=5). Patients with symptoms of hormone excess were explicitly included in five studies and confirmed hormone excess following imaging was identified in around a third of participants (mean 36%, range 0–88%; *n*=11 studies). Mean tumor size ranged from 19mm (42) to 68mm (55).

### Study quality

The vast majority (84%) of studies were at high or unclear risk of bias across all quality domains assessed (Fig. 1A and Supplementary Figs 1, 2, 3). A third of studies (*n*=12) only included participants selected for adrenalectomy and therefore at higher risk of malignancy, and four adopted a case–control type approach with separate selection of those with confirmed malignancy and benign disease (33, 39, 49, 53). PET (Supplementary Fig. 3) and MRI evaluations (Supplementary Fig. 2) were at particular risk of bias due to retrospective selection of the diagnostic threshold (in 8/9 and 6/15 evaluations, respectively), potentially leading to inflated estimates of test accuracy. Test interpretation could have been influenced by the same observer interpreting more than one imaging test in the same study (affecting 14 of 40 evaluations). Test interpretation was blinded to the reference standard diagnosis in around half of all test evaluations (52%; 21/40) and differential verification was present in 62% (23/37). More than half of studies used an inadequate reference standard either due to the use of biopsy rather than full excision of malignant masses (*n*=15, 40%) or imaging follow-up of <12 months (*n*=6, 16%). Concerns around the applicability of study results were high (*n*=8) or unclear (*n*=17) due to varying or unclear indications for imaging in the included populations and due to the evaluation of a new threshold, not previously assessed in other studies (present or unclear in 21 of 40 test evaluations).

### Results according to clinical pathway

Poor reporting of the clinical pathways leading to the conduct of the imaging tests resulted in exclusion of 19 of 37 eligible studies from analysis (described in Supplementary Table 3). Characteristics of the 18 studies eligible for analysis are provided according to clinical pathway in Table 2 and results of test performance are reported in Table 3, with raw data for all test evaluations provided in Supplementary Table 4.

**Table 2:** Characteristics of the 18 studies eligible for meta-analysis.

				**Exclusions**										
**Reference**	**Index test(s)**	**Study design**	**Population**	**HU (HU)**	**Size (mm)**	**Other**	**Pat./Les. (*N*)**	**I/KM/S (%)**	**HE (%)**	**Reference standard (%)**	**Dis *n*; %**	**No. ACC**	**No. mets**	**Threshold for malignancy**
Studies investigating incidentally detected tumors (*n*=7)														
Angelelli (2013)	CT	BPCP	Imaging series, ≥50% incidental	No	<10, >120	Cysts, myelolipoma	50/50	74/0/26	NR	42/58/0	28; 56%	7	13	1. >10 HU2. APW <60% OR RPW <40% at 10′3. APW <60% OR RPW <40% at 15′
Marin (2012)	MRI	NCR	Imaging series, ≥90% incidental	No	<10	No	59/66	100/0/0	NR	35/55/11	17; 26%	5	11	1. SII ≤23% (OP/IP dataset)‖
Maurea (2004)	MRI	NCP	Imaging series, ≥50% incidental	NR	NR	Functioning masses; Pheos (*n*=4) excluded by Bham team	30/30	66/33/0	0	63/37/0	8; 31%	4	3	1. ALR – qualitative*2. SI – qualitative‡
Nunes (2010)	PET	WPCR	Imaging series, ≥50% incidental	<10	NR	Pheos; prior cancer; ACC on CT; eventual washout of contrast >50% on CT	23/23	65/0/35	43	100/0/0	3; 13%	2	0	1. ALR SUV_max_ >1.6§2. SUV_max_ >3.4
Sandra-segaran (2011)	MRI	NCR	Imaging series, ≥50% incidental	No	<10	Myelolipoma; cysts; artifacts on diffusion weighted imaging; lack of adequate reference	48/49	69/31/0	2	38/63/0	12; 24%	1	9	1. ASR≥62 (ADC)¶2. SII ≤ 23% (ADC)‖
Tessonier (2008)	PET	WPCP	Imaging series, ≥90% incidental	<10	No	Functioning masses; washout on delayed enhanced CT, decrease of signal intensity on CS MRI	37/41	100/0/0	0	71/29/0	12; 29%	3	4	1. ALR SUV_max_ >1.8§2. SUV_max_ >3.28
Vilar (2008)	CT	NCR	Imaging series, ≥90% incidental	NR	NR	None reported	52/52	100/0/0	25%	38/40/17	13; 25%	2	5	1. >10 HU
Studies investigating tumors in participants with current or prior non-adrenal malignancy (n=11)														
Burt (1994)	MRI	NCP	Operable NSCLC, ≥90% known malignancy	NR	No	None reported	27/27	0/100/0	NR	100/4/0	4; 16%	0	5	1. ALR qualitative*
Choi (2013)	CT	WPCR	Imaging series, ≥90% known malignancy	No	No	All diagnoses other than adenoma and metastasis	36/40	0/100/0	NR	100/30/0	19; 48%	0	19	1. >10 HU2. APW at 15′ <60%3. RPW at 15′ <40%
Del Moral (2010)	PET–CT	NCR	Imaging series, ≥50% known malignancy	NR	NR	Symptomatic tumors; Contraindications to PET;	15/15	0/53/47	NR	87/13/0	11; 73%	3	5	1. ALR SUV_max_ >1.8§2. SUV_max_ >6
Frilling (2004)	CT	WPCP	Adrenalectomy series, ≥90% known malignancy	No	≤60	Evidence of extra-adrenal tumor spread	42/44	0/100/0	0	100/0/0	31; 70%	0	31	>10HU
Kunik-owska (2014)	PET-CT	WPCR	Imaging series, ≥90% known malignancy	No	No	Functioning masses	85/104	0/100/0	NR	100/0/0	32; 31%	1	30	1. ALR SUV_max_ >1.53§2. SUV_max_ >5.2
Lang (2015)	PET-CT	NCR	Adrenalect-omy series, ≥90% known malignancy	No	No	Functioning masses; no clinical suspicion of metastasis (based on CT findings)	39/39	0/100/0	0	100/0/0	29; 74%	0	28	1. ALR SUV_max_ >1.29§2. SUV_max_ >3.7
Mc-Nicholas (1995)	CTMRI	WPCP	Imaging series, ≥90% known malignancy	NR	<10	Pheos	33/37	0/100/0	NR	51/46/0	19; 51%	0	18	CT: >10HUMRI: ASR ≥75¶
Porte (1999)	CTMRI	WPCP	Operable NSCLC, ≥90% known malignancy	NR	<10	Pheos	32/32	0/100/0	NR	100/44/0	18; 56%	0	18	CT: >10HUMRI: ALR qualitative†
Ream (2014)	MRI	NCR	Imaging series, ≥50% known malignancy	No	<8	Myelolipoma; cysts; non-standardized imaging protocol; lack of adequate reference	36/37	NR/78/NR	NR	19/76/5	10; 28%	0	8	1. ALR >0.674††2. ASR >64.1‡‡3. AMR >70.7§§
Schwartz (1995)	MRI	NCP	Biopsy referrals, ≥90% known malignancy	NR	NR	None reported	68/68	0/100/0	NR	71/29/0	23; 34%	NR	NR	1. ALR ≥1.5**2. ASR ≥55¶
Uemura (2012)	CT	WPCR	Imaging series, ≥90% known malignancy	NR	NR	Grades 4 or 5 disease; bleeding tendency and coagulopathy	12/16	0/100/0	NR	93/0/7	6; 40%	0	6	1. >10HU

ACC, adrenocortical carcinoma; BPC, between-person comparison (multiple index tests evaluated in partial study population); APW, absolute percentage washout; ADC, apparent diffusion coefficient; ALR, adrenal to liver ratio; ASR, adrenal to spleen ratio; AMR, adrenal to muscle ratio; ASR, adrenal to spleen ratio; CS, chemical shift; Excl, exclusion; HU, Hounsfield units; IP, in-phase; METS, metastases; NC, non-comparative study; NR, not reported; NSCLC, non-small cell lung cancer; OP, opposed phase; P, prospective data collection; R, retrospective data collection; RPW, relative percentage washout; SI, signal intensity; SII, signal intensity index; SUV_max_, maximum standardized uptake value; WPC, within-person comparison (multiple index tests evaluated in all study participants).

* Masses considered to be malignant if their signal was more intense than liver signal

† Masses considered to be metastases if their signal was more intense than liver signal and inferior to kidney signal

‡ Masses considered to be malignant if no loss of signal intensity observed on chemical shift

§ ALR SUV_max_, ratio of SUV_max_ in the adrenal gland compared with the liver.

Formulae for calculating quantitative thresholds:

‖ Signal intensity index =[(SI adrenal IP) – (SI adrenal OP)] / (SI adrenal IP)

¶ MRI adrenal to spleen ratio=(SI adrenal OP/SI Spleen OP)/(SI adrenal IP/SI spleen IP)

** MRI adrenal to liver ratio=SI adrenal/SI liver

†† MRI adrenal to liver ratio=[(SI adrenal OP/SI liver OP)/(SI adrenal IP/SI liver IP)] – 1) × 100%

‡‡ MRI adrenal to spleen ratio=[(SI adrenal OP/SI spleen OP)/(SI adrenal IP/SI spleen IP)] – 1)×100%

§§ MRI adrenal to muscle ratio=[(SI adrenal OP/SI muscle OP)/(SI adrenal IP/SI muscle IP)] – 1)×100%].

**Table 3:** Test performance according to clinical pathway. Studies focusing on truly incidentally discovered adrenal masses (incidentaloma pathway) vs studies on adrenal masses discovered during follow-up monitoring for extra-adrenal malignancy (follow-up from previous malignancy pathway).

	≥ 50%*			≥90%**		
	**Studies (*n/N*)**	**Sensitivity (95% CI)**	**Specificity (95% CI)**	**Studies (*n/N*)**	**Sensitivity (95% CI)**	**Specificity (95% CI)**
Incidentaloma pathway						
CT non–contrast tumor density (>10HU)	2 (41/102)	100% (91–100%)	72% (60–82%)	1 (13/52)	100% (75–100%)	72% (55–85%)
CT contrast enhanced washout (combination at 10min)	1 (14/25)	93% (68–100%)	100% (69–100%)	0	–	–
CT contrast enhanced washout (combination at 15min)	1 (13/25)	100% (75–100%)	92% (62–100%)	0	–	
MRI adrenal-liver ratio (1.5Tesla only)	1 (8/26)	100% (63–100%)	44% (22–69%)	0	–	–
MRI adrenal-spleen ratio (1.5Tesla only)	1 (12/49)	58% (28–85%)	86% (71–95%)	0	–	–
MRI loss of signal intensity (1.5Tesla only)	2 (20/75)	86% (31–99%)	85% (73–93%)	0	–	–
PET ALR SUV_max_	2 (15/64)	100% (78–100%)	96% (57–100%)	1 (12/41)	100% (74–100%)	100% (88–100%)
PET SUV_max_	2 (15/64)	93% (65–99%)	73% (59–84%)	1 (12/41)	92% (62–100%)	72% (53–87%)
Follow-up from previous malignancy pathway						
CT non–contrast tumor density (>10HU)	5 (93/168)	93% (79–98%)	71% (38–91%)	5 (93/168)	93% (79–98%)	71% (38–91%)
CT contrast enhanced washout (absolute at 15min)	1 (19/40)	16% (3–40%)	86% (64–97%)	1 (19/40)	16% (3–40%)	86% (64–97%)
CT contrast enhanced washout (relative at 15min)	1 (19/40)	16% (3–40%)	95% (76–100%)	1 (19/40)	16% (3–40%)	95% (76–100%)
MRI adrenal-liver ratio (1.5Tesla only)	3 (37/129)	89% (74–96%)	60% (21–89%)	2 (27/93)	92% (55–99%)	39% (21–60%)
MRI adrenal-spleen ratio (1.5Tesla only)	3 (52/142)	99% (69–100%)	84% (72–91%)	2 (42/105)	100% (92–100%)	79% (68–88%)
MRI adrenal-muscle ratio (1.5Tesla only)	1 (10/37)	90% (55–100%)	93% (76–99%)	0	–	–
MRI loss of signal intensity (1.5Tesla only)	1 (10/37)	90% (55–100%)	85% (66–96%)	0	–	–
PET ALR SUV_max_	2 (45/117)	82% (41–97%)	96% (76–99%)	1 (34/102)	94% (80–99%)	94% (86–98%)
PET SUV_max_	3 (72/156)	84% (62–94%)	90% (71–97%)	2 (61/141)	90% (80–96%)	87% (78–93%)

ALR SUV_max_, ratio of SUV_max_ in the adrenal gland compared with the liver; HU, Hounsfield units; *n*, number of cases; *N*, total population; PET, positron emission tomography; SUV_max_, maximum standardized uptake value.

* refers to ≥50% with incidentaloma in studies in the incidentaloma pathway and ≥50% with current or prior non-adrenal malignancy in the follow-up from previous malignancy pathway; **refers to ≥90% with incidentaloma in studies in the incidentaloma pathway and ≥90% with current or prior non-adrenal malignancy in the follow-up from previous malignancy pathway.

### Test performance in the investigation of incidentally detected tumors

Seven studies presented data on test performance (two for CT (27, 30), three for MRI (28, 31, 46), and two for PET-CT (29, 61)) in patient groups presenting with more than 50% (and two with >90%) incidentally detected tumors. Two studies evaluating tumor density >10HU on non-contrast CT (27, 30), and one evaluating CT contrast-enhanced washout tests (27) showed high sensitivity and specificity. Only two (28, 31) of the three studies of MRI used 1.5 Tesla machines and reported slightly lower sensitivity and specificity than CT for measures of adrenal-liver and adrenal-spleen ratios and loss of signal intensity. The performance of PET for ALR and SUV_max_ measures was no better than CT.

The data suggest that CT density >10HU has high sensitivity for the detection of malignancy, the 95% CI suggesting that this is above 90%. However, all other estimates of test performance are based on small numbers of studies with few patients, and 95% CIs are notably wide, indicating uncertainty in test performance for all other imaging markers. It is not possible to discern from the available data whether any test performs adequately or better than alternative tests.

### Test performance in the investigation of tumors in participants with current or prior non-adrenal malignancy

Eleven studies presented data on test performance (five for CT (7, 33, 34, 35, 37), five for MRI (32, 34, 35, 36, 60), and three for PET-CT (8, 38, 62)) in patient groups presenting with more than 50% (and 9 with >90%) tumors detected in patients undergoing imaging following previous non-adrenal malignancy. The five studies evaluating CT density >10HU on non-contrast CT (7, 33, 34, 35, 37) showed high sensitivity (93%) but variable specificity; CT contrast-enhanced washout tests were only reported in one study (33), which showed very low sensitivity (16%). Four (32, 34, 36, 60) of the five studies of MRI used 1.5 Tesla machines and reported high ­sensitivity (89–99%) for measures of adrenal-liver, adrenal-spleen, adrenal-muscle ratios and loss of signal intensity. Specificity varied (60–93%) but was high for most MRI measures. The performance of PET was similar to MRI for ALR and SUV_max_ measures.

Although more studies had evaluated CT, MRI, and PET in the pathway for follow-up of known malignancy than for incidentally discovered adrenal lesions, estimates of test performance are still based on too small numbers of studies to be able to discern whether any test performs adequately or better than alternative tests from the ­available data.

## Discussion

Our main finding cautiously suggests that in patients without known extra-adrenal malignancy, a non-contrast CT tumor density of 10HU is a diagnostically relevant cut-off, albeit based only on data from two small studies. The sensitivity of >10HU for detecting malignancy was high (100%; 95% CI: 91, 100%), however, the specificity was poor. Conversely, this means that an incidentally discovered adrenal mass with a non-contrast CT tumor density of ≤10HU is unlikely to be malignant. Tumor density ≤10HU was less conclusive for ruling out malignancy in patients with a history of extra-adrenal malignancy, however, with a pooled false-negative rate of 7%, although CIs were wide. With positive predictive values for detection of malignancy in the order of 70–80% in both populations, a considerable number of adrenal masses with tumor density >10HU are likely to be benign. These and all other pooled estimates have such wide CIs that no further conclusions can be drawn regarding the accuracy of imaging tests for the detection of malignancy in incidentally discovered adrenal masses.

Possible clinical explanations for this uncertainty include variability in the lipid content of adenomas, tissue heterogeneity, small size of metastatic lesions, or differences in selecting regions of interest forHU measurement. However, most of the uncertainty is due to small numbers of eligible studies and hence results from few patients available for analysis. Despite the availability of a significant number of studies addressing imaging characteristics in patients with an adrenal mass, more than 90% of full-text papers retrieved had to be excluded. Many had small sample sizes, mixed populations, inadequate reporting on imaging techniques and thresholds, as well as unacceptable reference standards for both malignant and benign masses. Even with our stringent eligibility criteria, included studies were characterized by heterogeneity in study populations, imaging tests and thresholds, and reference standards as well as poor methodological quality. Given differences in patient spectrum according to the indication for adrenal imaging and the potential impact on accuracy (63, 64, 65), our meta-analysis was further restricted to studies where a majority of participants had either incidentaloma or were undergoing imaging due to known malignancy, leading to the exclusion of another 50% of included studies. Heterogeneity in study conduct and poor methodological quality remained, further contributing to the lack of certainty in pooled estimates.

Our findings are disappointingly consistent with another systematic review of the literature on tests for adrenal incidentaloma published almost 15 years ago (66). Observed heterogeneity in tests and populations meant that no meta-analysis was undertaken and no clear conclusions could be drawn (66). Almost three-quarters (27/37) of the studies in our review were published in the interim period; however, methodological and reporting quality have not improved sufficiently to allow any new conclusions to be drawn. A more recent meta-analysis of FDG-PET (67) applied considerably less stringent inclusion criteria compared with our review, thereby including more studies (*n*=21); however, highly heterogeneous data limited the conclusions that could be drawn.

Our findings of poor quality and reporting of test accuracy studies are similar to findings from other fields (68, 69, 70). Introduction of the Standards for Reporting Diagnostic Accuracy (STARD) statement (71) has only led to small improvements in reporting (72) and our results indicate that greater awareness is required of methodological considerations in the design and delivery of multicenter studies in this field, as in many others, to improve reporting.

The strengths of this review include an in-depth comprehensive literature search, a focused review question, and stringent predefined reference standard. The limitations were derived from the heterogeneity and low quality of included studies. Unclear definitions of study populations, various and often data-driven thresholds, as well as different techniques for the same imaging tests, limit the interpretation and generalization of results. The weak conclusions derived from this systematic review and meta-analysis should be interpreted in relation to the low volume and poor quality of included studies (Fig. 1B).

Our results do not suggest that current imaging practice is inappropriate: small study numbers prevent us from providing substantive evidence to either support current practice or to prompt a need for a change in imaging practice. We suggest further studies are needed to answer the following key questions:

1. Do adrenal lesions with unenhanced CT tumor density ≤10HU need additional imaging, in particular in patients with a history of extra-adrenal malignancy?
2. What is the best second-line imaging study that would accurately diagnose (or exclude) a malignant adrenal mass?
3. What additional factors influence decisions on imaging choice? (patient preference, radiation risks, costs)
4. How much tumor growth, and over what period of time, is indicative of a malignant adrenal mass?

In addition, future studies should include the systematic evaluation of alternative testing approaches and detailed analysis of health economics impact. All these questions can only be answered with larger multicenter studies, with prospective recruitment of consecutive series of participants in appropriately defined clinical pathways, and imaging test interpretation blinded to the reference standard diagnosis and to the result of any other imaging tests. Diagnostic thresholds for determining benignity or malignancy must be prespecified to avoid data-driven threshold selection and overestimation of test accuracy. The reliance on a histological reference standard leads to study populations with a high pretest probability of malignancy, however, imaging follow-up of those with indeterminate imaging characteristics needs to be long enough to ensure that malignant masses are not missed. Centralized radiological and pathology review would further help to strengthen the results. Future investigators must also meet the updated STARD recommendations (20) so that study conduct and quality can be judged appropriately.

In conclusion, current evidence on imaging tests and cut-offs in diagnosis of incidentally discovered adrenal mass is highly heterogeneous and disappointingly poor. Not surprisingly, many patients with adrenal incidentaloma undergo repeated multimodal imaging and even unnecessary adrenalectomy. With adrenal incidentalomas detected on 1 in 20 (1, 2, 3) of an ever-increasing number of cross-sectional abdominal imaging studies performed every year, the potential economic and health impact of unnecessary procedures and interventions could be significant. In this era of evidence-based medicine, and with advances in our understanding of optimal diagnostic test accuracy study design and study synthesis, it is incumbent on the medical community to provide a solid evidence base to underpin imaging practice in this field. Areas of uncertainty especially include second-line testing for indeterminate adrenal masses and larger adrenal masses, with very limited data on CT washout, MRI, and PET-CT. Further well-designed studies are needed to establish performance and health economic impact of imaging in patients with incidentally discovered adrenal masses.

This meta-analysis has informed the ESE-ENSAT Clinical Guidelines on the management of adrenal incidentalomas (73).

## Supplementary data

This is linked to the online version of the paper at http://dx.doi.org/10.1530/EJE-16-0461.

## Declaration of interest

W A holds a patent on rapid diagnosis of adrenal malignancy by urine steroid metabolomics. All other authors have nothing to disclose.

## Funding

This work was supported by a Mayo Foundation Scholarship (to I B), the Wellcome Trust (Clinical Research Training Fellowship 101671, to V C), and the European Union (Seventh Framework Program; FP7/2007-2013, Grant agreement 259753, ENSAT-CANCER, to W A). J D is a National Institute for Health Research Senior Investigator. The funding agencies had no role in study design, data collection, analysis, or interpretation of this work.

## Author contribution statement

Data extraction and quality assessment: J D, I B, L F R, V C, C D; Data analysis: J D, L F R, S B, J J D; Manuscript writing: J D, I B, L F R, A S, P G, M F, J J D, W A; Expert review and advice: A S, P G, J J D, W A.
